# 

**Published:** 1881-07

**Authors:** 


### BOOKS AND PAMPHLETS RECEIVED.

A Treatise on Bright's Disease and Diabetes. By James Tyson, M.D.

A Treatise on Albuminuria. By W. Howship Dickinson, M.D.

A Treatise on the Materia Medica and Therapeutics of the Skin. By Henry G. Piffard, M.D.

A Treatise on The Continued Fevers. By James C. Wilson, M.D.

Cyclopædia of the Practice of Medicine. By Dr. H. von Ziemssen, Vol. IX.

Medical Electricity: A Practical Treatise on the Applications of Electricity to Medicine and Surgery. By Roberts Bartholow, M.D.

The Transactions of the American Medical College. Vol. XXXI, 1880.

On the Antagonism Between Medicines and Between Remedies and Disease. By Robert Bartholow, M D., LL.D.

Dr. Paul Borners Reich's Medicinal Kalender fur Deutschland auf das Jar, 1881. Part II.

Transactions of the Illinois State Medical Society, for 1878.

Anatomical Plates. By Ambrose L. Ranney, M.D.

How we Feed the Baby to Make Her Healthy and Happy, with Health Hints. By C. E. Page, M.D.

Illinois State Medical Register for 1877–8. By D. W. Graham, M.D.

The Diet Cure: An Essay on the Relations of Food and Drink to Health, Disease and Cure. By T. L. Nichol, M.D.

An Introduction to Pathology and Morbid Anatomy. By T. H. Green, M.D.

The Metric System in Medicine. By Oscar Oldberg, Phar. D.

Lectures on the Diseases of the Nervous System, especially in Women. By S. Weir Mitchell, M.D.

What Every Mother Should Know. By Edward Ellis, M.D.

The Normal Temperature of the Head. By J. S. Lombard, M.D.

The Diseases of Children; a Practical and Systematic work for Practitioners and Students. By William Henry Day, M.D.

Medical Register and Directory, being a Treatise on the Diseases and Surgery of the Mouth, Jaws and Associate Parts. By James E. Garretson, M.D.

Atlas of Human Anatomy, containing 180 large plates, arranged according to Drs. Oesterreicher and Erdl, from their original designs from nature and those of the greatest anatomists of modern times, with full explanatory texts. By J. A. Jeancon, M.D. In 12 numbers, from Parts 31—42.

Supplement to Zeimssen's Cyclopædia of the Practice of Medicine. By George L. Peabody, M.D.

A Medical Formulary, based on the United States and British Pharmacopœias, together with numerous French, German and Unofficinal Preparations. By Laurence Johnson, M.D.

The Principles of Myodynamics. By J. S. Wright, M.D.

Hygiene and Treatment of Catarrh. Therapeutic and Operative Measures for Chronic Catarrhal Inflammation of the Nose, Throat and Ears. Part II. By Thomas F. Rumbold, M.D.

The Hygiene and Treatment of Catarrh. Part I—Hygiene and Sanative Measures. Part II—Therapeutic Measures. By Thos. F. Rumbold, M.D.

Introduction to the Study of Indian Languages, with Words, Phrases and Sentences to be Collected. By J. W. Powell.

---

ALUMNI PRIZE.—The Prize of the Alumni Association of the College of Physicians and Surgeons of New York, of $500, for an original essay on some subject connected with medicine or surgery, is open only to the competition of the Alumni of the College of Physicians and Surgeons.

The conditions upon which the prize will be awarded are as follows:

1. The subject is left to the option of the competitor.

2. The essay must present sufficient original, experimental or clinical observation to make it a useful contribution to medical knowledge.

3. The essay, designated by a motto, must be sent to a member of the Committee on Prize Essays, accompanied by a sealed envelope, inscribed with the same motto, and containing the name and address of the author, on or before April 1, 1882.

ALBERT H. BUCK, M.D., 109 Madison av.
CHARLES MCBURNEY, M.D., 40 West 36th street.
GEORGE L. PEABODY, M.D., 57 West 38th street.

*Committee.*

SOCIETY MEETINGS.

Chicago Medical Society—Mondays, July 4-18.
West Chicago Medical Society—Mondays, July 11-25.
Biological Society—Wednesday, July 6.

CLINICS.

MONDAY.

Eye and Ear Infirmary—2 p. m., Ophthalmological, by Prof. Holmes; 3 p. m., Otological, by Prof. Jones.

Mercy Hospital—2 p. m., Surgical, by Prof. Andrews.

Woman's Medical College—2 p. m., Dermatological and Venereal, by Prof. Maynard; 3 p. m., Diseases of the Chest, Prof. Ingals.

TUESDAY.

Rush Medical College—3 p. m., Dermatological and Venereal, by Prof. Hyde.

Cook County Hospital—2 to 4 p. m., Medical and Surgical Clinics.

Mercy Hospital—2 p. m., Medical, by Prof. Quine.

WEDNESDAY.

Chicago Medical College—2 p. m., Eye and Ear, by Prof. Jones.

Rush Medical College—2 p. m., Medical, by Dr. Bridge; 3 p. m., Ophthalmological and Otological, by Prof. Holmes; 3:30 to 4:30 p. m., Diseases of the Chest, by Dr. E. Fletcher Ingals.

THURSDAY.

Chicago Medical College—2 p. m., Gynæcological, by Prof. Jenks.

Rush Medical College—2 p. m., Diseases of Children, by Dr. Knox; 3 p. m., Diseases of the Nervous System, by Prof. Lyman.

Eye and Ear Infirmary—2 p. m., Ophthalmological, by Dr. Hotz.

Woman's Medical College—3 p. m., Surgical, by Prof. Owens.

FRIDAY.

Cook County Hospital—2 to 4 p. m., Medical and Surgical Clinics.

Mercy Hospital—2 p. m., Medical, by Prof. Davis.

SATURDAY.

Rush Medical College—2 p. m., Surgical, by Prof. Gunn; 3 p. m., Orthopœdic, by Prof. Owens.

Chicago Medical College—2 p. m., Surgical, by Prof. Isham; 3 p. m., Neurological, by Prof. Jewell.

Woman's Medical College—11 a. m., Ophthalmological, by Prof. Montgomery; 2 p. m., Gynæcological, by Prof. Fitch.

Daily Clinics, from 2 to 4 p. m., at the Central Free Dispensary, and at the South Side Dispensary.

